# Supplementary material for: Genetic inactivation of SARM1 axon degeneration pathway improves outcome trajectory after experimental traumatic brain injury based on pathological, radiological, and functional measures
Source: Acta Neuropathol Commun. 2021 May 17;9:89. doi: 10.1186/s40478-021-01193-8 (PMC8130449; doi:10.1186/s40478-021-01193-8)
Supplement: Supplementary file 1 — Additional file 1. [file 40478_2021_1193_MOESM1_ESM.pdf]

Genetic inactivation of SARM1 axon degeneration pathway improves outcome trajectory after experimental traumatic brain injury based on pathological, radiological and functional measures

Donald V. Bradshaw Jr.[1,2], Andrew K. Knutsen[3,4], Alexandru Korotcov[3,4], Genevieve M. Sullivan[2], Kryslaine L. Radomski[2,4], Bernard J. Dardzinski[3,4] *, Xiaomei Zi[2], Dennis P. McDaniel[5], and Regina C. Armstrong[1,2,4 #]

[1]Graduate Program in Neuroscience,
[2]Department of Anatomy Physiology and Genetics
[3]Department of Radiology and Radiological Sciences,
[4]Center for Neuroscience and Regenerative Medicine,
[5]Biomedical Instrumentation Center,
F. Edward Hebert School of Medicine, Uniformed Services University of the Health Sciences, Bethesda, MD 20814 USA

#Corresponding Author:
Regina C. Armstrong, PhD
Department of Anatomy, Physiology and Genetics
Uniformed Services University of the Health Sciences
4301 Jones Bridge Rd., Bethesda, MD 20814
E-mail: regina.armstrong@usuhs.edu

*Current address:
Center for Scientific Review
National Institutes of Health, Bethesda, MD

Supplemental Figure S1: Righting reflex times after sham or TBI surgical procedure in *Sarm1* WT and *Sarm1* KO mice. Post-surgical righting reflex times were measured as the time interval from the end of anesthesia until mice returned to the upright position. Righting time is shown for all mice used in experiments. Prolonged righting reflex times are an indicator of TBI severity that serve as a measure of arousal that estimates loss of consciousness after TBI or sham procedures. Righting reflex time did not differ between male and female littermates, so the sexes were combined for analysis by genotype. Righting reflex time was significantly prolonged after TBI as compared to the sham procedure for *Sarm1* WT ($p < 0.0001$) and *Sarm1* KO ($p < 0.001$) mice without a difference based on genotype ($p = 0.8287$; Two-way ANOVA; $F(1,68) = 183$).

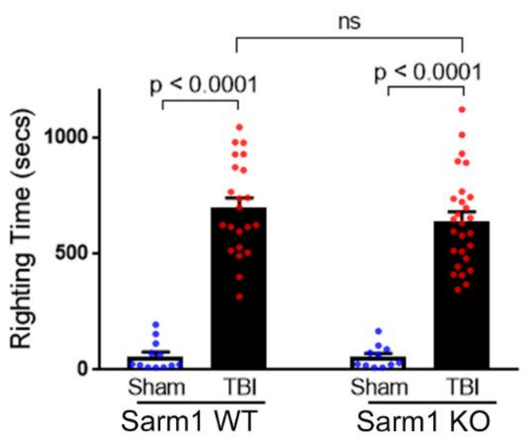

Supplemental Figure S2. TBI reduces white matter integrity and produces significant corpus callosum (CC) atrophy in C57BL/6 mice. A cross-sectional *in vivo* MRI study used male C57BL/6J mice between 9-10 weeks post-TBI/sham. After scanning, mice were perfused for tissue analysis at 10 weeks post-TBI/sham. **A-B:** CC volume changes were calculated as the change in volume compared to a registered template image of all animals in the study. Seven ROIs were manually drawn on coronal images (125 µm thickness) encompassing the rostro-caudal CC over the lateral ventricle and underneath the impact site at bregma (A). TBI significantly reduced the mean CC volume, as compared to the sham procedure (B) **C-E:** Post-imaging neuropathology validated CC atrophy after TBI. Myelinated fibers were immunolabeled for myelin oligodendrocyte glycoprotein (MOG; red) and cellular distribution was detected using nuclear counterstain (DAPI; blue) (C, D). The CC borders (dashed lines) were evident by the pattern of myelinated fibers oriented in the medial-lateral direction in the CC in contrast to the myelinated fibers of the cingulum (Cg) that align in the rostro-caudal (i.e. anterior-posterior) direction. Double headed arrows (C, D) show examples of sites for CC width measurement for quantification (E). **F-J:** MRI diffusion tensor imaging (DTI) illustrates fractional anisotropy signal in the CC (white arrows) and adjacent regions in direction encoded color maps (F, G). Colors represent fiber directions as red (medial-lateral), blue (anterior-posterior), and green (superior-inferior). TBI significantly reduced the CC fractional anisotropy (FA; H) which was driven by reduced axial diffusivity (AD; I) and increased radial diffusivity (RD; J). MRI n = 12 per condition; male C57BL/6J mice (RRID:IMSR_JAX:000664), Jackson Laboratories, Bar Harbor, ME). Neuropathology n = 6 per condition randomly selected from the MRI cohort. Student's *t*-test.

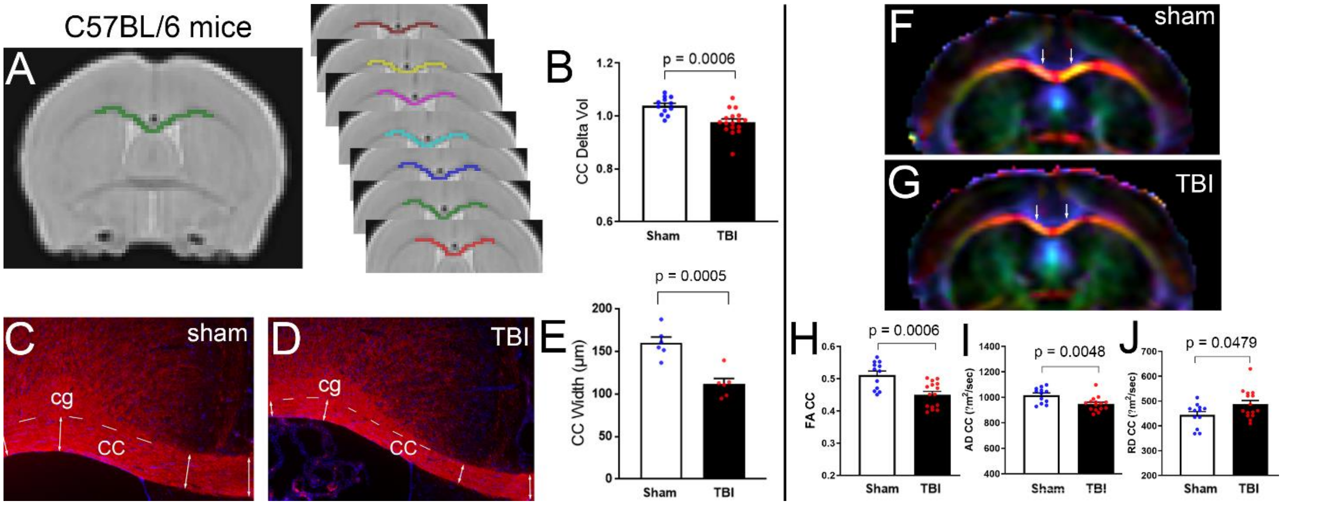

Supplemental Figure S3. TBI causes a chronic stage motor learning deficit in C57BL/6J mice. Miss-step wheels, which have been shown to engage the CC and be sensitive to myelination status, were used to assess chronic stage changes between sham and TBI mice. The wheels have irregularly spaced rungs to assess motor skill learning (week 1) followed by a plateau phase (week 2) that tests bilateral sensorimotor function. TBI mice show a significant decrease in average running velocity compared to sham during the learning phase (week 1). There was no statistically significant difference between TBI and sham groups during the plateau phase of the assay (week 2). Wheels n = 9 per condition; male C57BL/6J mice (RRID:IMSR_JAX:000664), Jackson Laboratories, Bar Harbor, ME). Two-way repeated measures mixed effects model ANOVA; Learning Phase: Time $p < 0.0001$, $F(2.640, 40.93) = 84.41$; Injury $p = 0.0374$, $F(1,16) = 5.151$; Time x Injury $p = 0.0299$, $F(6, 93) = 2.457$. Plateau Phase: not significant.

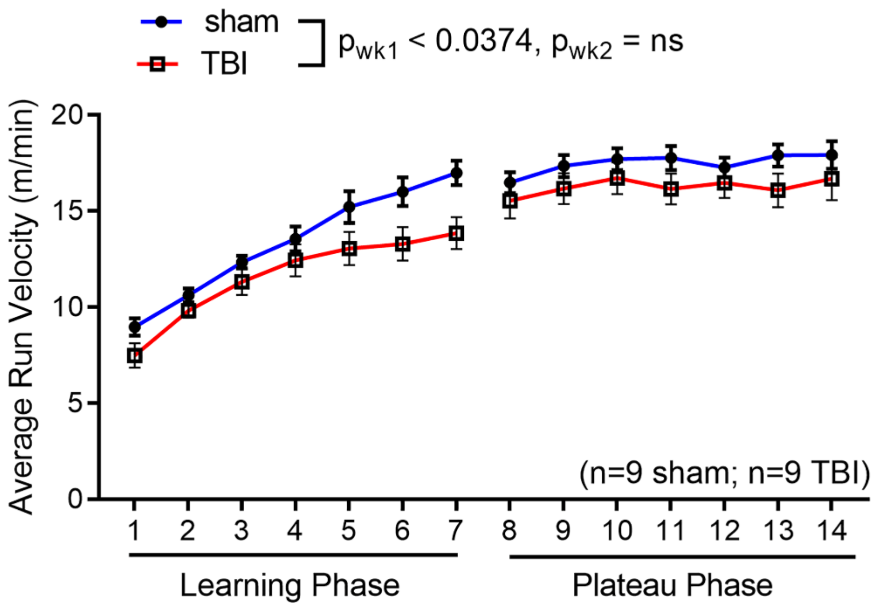

Supplemental Figure S4. TBI does not induce a late-stage social deficit despite CC atrophy in C57BL/6J mice. In their 8th week post injury mice were tested using a social interaction assay to measure propensity to engage in social behavior. For five minutes mice freely explore a three chamber apparatus with one chamber containing a novel, unfamiliar mouse in a wire carrier and another possessing an empty wire carrier while their time spent interacting with each carrier is

measured. Our previous study showed that CC pathology in an experimental model of chronic demyelination induces a social deficit [1]. This study looked to determine if a social deficit, that was not present at more acute time points after single impact TBI [2], develops at a chronic time point. Both TBI and sham mice spent a significantly greater amount of time interacting with the carrier containing the unfamiliar mouse compared to the empty carrier (**A**). There was no statistically significant difference in interaction time between sham and TBI groups. At 10 weeks post TBI mice were sacrificed for neuropathological evaluation of CC width. TBI mice exhibit significant late-stage CC atrophy compared to sham (**B**). The absence of a social deficit at 8 weeks post TBI despite ongoing, chronic atrophy of the CC resulted in the omission of the social interaction assay from our study evaluating the therapeutic benefit of *Sarm1* genetic deletion. Social Interaction n = 12 per condition; male C57BL/6J mice (RRID:IMSR_JAX:000664), Jackson Laboratories, Bar Harbor, ME). Neuropathology n = 6 per condition randomly selected from the MRI cohort. Social interaction: Two-way ANOVA; $F(1,44) = 0.2116$. Neuropathology: Student's *t*-test.

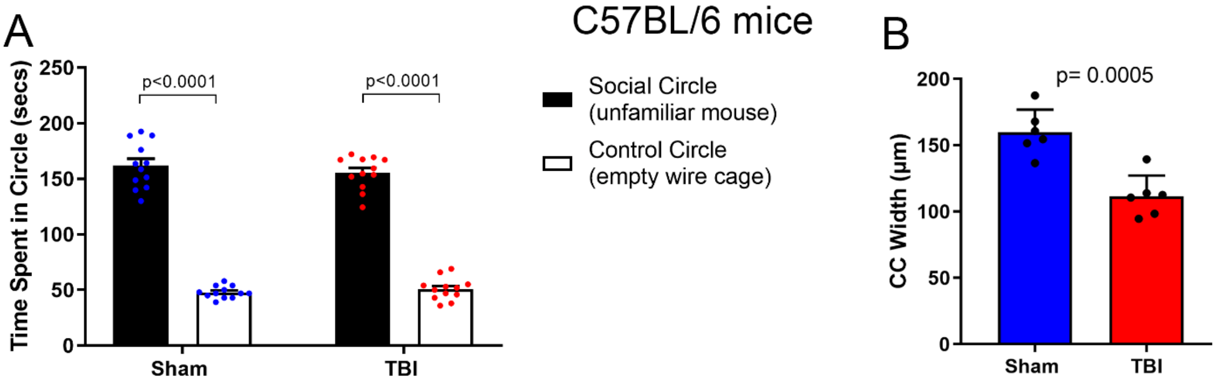

Table S1 ***Statistics for Electron Microscopy study at 10 weeks post-injury or sham procedure.***
Two-way ANOVA with Holm-Sidak's multiple comparisons test for the adjusted p-values

**Electron Microscopy**	**Main; F, p-value**		**Adjusted p-value**	
CC width	interaction	F (1,28) = 0.4778; p = 0.4591	WT sham-TBI	***p = 0.0001***
	injury	F (1,28) = 41.50; p < 0.0001	KO sham-TBI	***p = 0.0014***
	genotype	F (1,28) = 10.92; p = 0.0026	Sham WT-KO	p = 0.0753
			TBI WT-KO	***p = 0.0256***
Intact myelinated axons	interaction	F (1, 29) = 22.64; p < 0.0001	WT sham-TBI	***p < 0.0001***
	injury	F (1, 29) = 48.70; p < 0.0001	KO sham-TBI	p = 0.3501
	genotype	F (1, 29) = 34.11; p < 0.0001	Sham WT-KO	p = 0.6461
			TBI WT-KO	***p < 0.0001***
Damaged axons	interaction	F (1, 28) = 11.41; p = 0.0022	WT sham-TBI	***p < 0.0001***
	injury	F (1, 28) = 220.9; p < 0.0001	KO sham-TBI	***p < 0.0001***
	genotype	F (1, 28) = 22.64; p = 0.0063	Sham WT-KO	p = 0.7676
			TBI WT-KO	***p = 0.0002***
Axons with abnormal mitochondria	interaction	F (1, 29) = 2.736; p = 0.1089	WT sham-TBI	***p = 0.0152***
	injury	F (1, 29) = 8.598; p = 0.0065	KO sham-TBI	p = 0.6181
	genotype	F (1, 29) = 0.6703; p = 0.4196	Sham WT-KO	p = 0.6181
			TBI WT-KO	p = 0.3000
De/unmyelinated axons	interaction	F (1, 29) = 12.64; p = 0.0013	WT sham-TBI	***p < 0.0001***
	injury	F (1, 29) = 28.66; p < 0.0001	KO sham-TBI	p = 0.3941
	genotype	F (1, 29) = 6.227; p = 0.0185	Sham WT-KO	p = 0.4675
			TBI WT-KO	***p = 0.0006***

Table S2 ***Statistics for MRI study.*** *Two-way RM ANOVA with Holm-Sidak's multiple comparisons*

Magnetic Resonance Imaging	Main; F, p-value		Adjusted p-value
CC volume	interaction	F (1,21) = 3.686; p = 0.0685	WT BL-10wk ***p = 0.0014***
	time	F (1,21) = 16.53; p = 0.0006	KO BL-10wk p = 0.1187
	genotype	F (1,21) = 0.04581; p = 0.8326	WT-KO BL p = 0.5277
			WT-KO 10wk p = 0.5270
Fractional Anisotropy	interaction	F (2, 46) = 2.780; p = 0.0725	WT BL-3D ***p = 0.0066***
	time	F (1.674, 38.51) = 33.63; p <0.0001	WT BL-10wk ***p = 0.0012***
	genotype	F (1, 23) = 1.208; p = 0.2831	WT 3D-10wk p = 0.6559
			KO BL-3D ***p = 0.0143***
			KO BL-10wk ***p < 0.0001***
			KO 3D-10wk ***p = 0.0002***
			BL WT-KO p = 0.3477
			3D WT-KO p = 0.2415
			10wk WT-KO p = 0.6749
Axial Diffusivity	interaction	F (2, 46) = 0.1019; p = 0.9033	WT BL-3D ***p = 0.0067***
	time	F (1.920, 44.15) = 15.35; p < 0.0001	WT BL-10wk ***p = 0.0001***
	genotype	F (1, 23) = 8.207; p =0.0088	WT 3D-10wk p = 0.9618
			KO BL-3D ***p = 0.0113***
			KO BL-10wk ***p = 0.0113***
			KO 3D-10wk p = 0.5266
			BL WT-KO ***p = 0.0224***
			3D WT-KO p = 0.0810
			10wk WT-KO p = 0.0810
Radial Diffusivity	interaction	F (2, 46) = 4.574; p = 0.0154	WT BL-3D p = 0.1052
	time	F (1.952, 44.89) = 22.84; p < 0.0001	WT BL-10wk ***p = 0.0374***
	genotype	F (1, 23) = 0.002206; p = 0.9629	WT 3D-10wk p = 0.4728
			KO BL-3D p = 0.1335
			KO BL-10wk ***p < 0.001***
			KO 3D-10wk ***p = 0.0008***
			BL WT-KO p = 0.7014
			3D WT-KO p = 0.4378
			10wk WT-KO p = 0.1344
Trace	interaction	F (2, 46) = 1.477; p = 0.2390	WT BL-3D p = 0.9758
	time	F (1.609, 37.01) = 2.550; p = 0.1019	WT BL-10wk p = 0.9756
	genotype	F (1, 23) = 2.813; p = 0.1071	WT 3D-10wk p = 0.9756
			KO BL-3D p = 0.3103
			KO BL-10wk p = 0.1024
			KO 3D-10wk p = 0.1024
			BL WT-KO p = 0.4486
			3D WT-KO p = 0.5880
			10wk WT-KO ***p = 0.0412***

Table S3 ***Statistics for immunohistochemistry studies at 10 weeks post-injury or sham procedure.*** Two-way ANOVA for genotype and injury effects with Holm-Sidak's to adjust for multiple comparisons. Unpaired *t*-test for comparing genotype in the traumatic brain injury condition among only MRI mice.

Immunohistochemistry	Main; F, p-value		Post hoc; adjusted p-value
MOG % CC labelling	interaction	F (1, 24) = 4.828; p = 0.0379	WT sham-TBI ***p < 0.0001***
	injury	F (1, 24) = 30.64; p < 0.0001	KO sham-TBI p = 0.1548
	genotype	F (1, 24) = 7.107; p = 0.0135	Sham WT-KO p = 0.7569
			TBI WT-KO ***p = 0.0002***
MOG % CC labelling MRI mice	t = 2.999, df = 15		TBI WT-KO ***p = 0.0090***
MOG CC width	interaction	F (1, 24) = 14.32; p = 0.0009	WT sham-TBI ***p < 0.0001***
	injury	F (1, 24) = 93.19; p < 0.0001	KO sham-TBI ***p = 0.0008***
	genotype	F (1, 24) = 15.02; p = 0.0007	Sham WT-KO p = 0.9517
			TBI WT-KO ***p < 0.0001***
MOG CC width MRI mice	t = 3.895, df=16		TBI WT-KO ***p = 0.0013***
GFAP % CC labelling	interaction	F (1, 23) = 12.24; p = 0.0019	WT sham-TBI ***p < 0.0001***
	injury	F (1, 23) = 51.75; p< 0.0001	KO sham-TBI p = 0.0514
	genotype	F (1, 23) = 13.76; p = 0.0012	Sham WT-KO p = 0.8856
			TBI WT-KO ***p = 0.0001***
IBA1 % CC labelling	interaction	F (1, 23) = 0.1041; p = 0.7499	WT sham-TBI ***p < 0.0001***
	injury	F (1, 23) = 69.10; p < 0.0001	KO sham-TBI ***p < 0.0001***
	genotype	F (1, 23) = 1.021; p = 0.3228	Sham WT-KO p = 0.6379
			TBI WT-KO p = 0.5724
IBA1+ cells in CC	interaction	F (1, 23) = 11.66; p = 0.0024	WT sham-TBI ***p < 0.0001***
	injury	F (1, 23) = 81.08; p < 0.0002	KO sham-TBI ***p = 0.0015***
	genotype	F (1, 23) = 8.045; p = 0.0094	Sham WT-KO p = 0.6924
			TBI WT-KO ***p = 0.0006***
IBA1+ resting microglia	interaction	F (1, 23) = 9.144	WT sham-TBI ***p < 0.0001***
	injury	F (1, 23) = 68.55	KO sham-TBI ***p = 0.0031***
	genotype	F (1, 23) = 4.739	Sham WT-KO p = 0.5628
			TBI WT-KO ***p = 0.0031***
IBA1+ activated microglia	interaction	F (1, 23) = 11.33	WT sham-TBI ***p < 0.0001***
	injury	F (1, 23) = 68.85	KO sham-TBI ***p = 0.0047***
	genotype	F (1, 23) = 9.924	Sham WT-KO p = 0.8820
			TBI WT-KO ***p = 0.0004***
βAPP+ axons/mm^2 in CC MRI mice	t = 5.315, df = 14		TBI WT-KO ***p = 0.0001***

Table S4 ***Statistics for Miss-step Wheel Studies at 8-10 weeks post-injury.*** *Statistical test noted in table.*

**Miss-step Wheel Studies**	**Main; F, p-value**		**Adjusted p-value**
**Average Run Velocity**		**WT TBI - KO TBI**	
Two-way ANOVA,	Week 1		Day 1 ***p = 0.0472***
Holm-Šídák's multiple	interaction	F (6, 132) = 0.6034; p = 0.7272	Day 2 p = 0.5275
comparisons test	day	F (6, 132) = 49.44; p < 0.0001	Day 3 p = 0.5275
	genotype	F (1, 22) = 3.320; p = 0.0821	Day 4 p = 0.5729
	subject	F (22, 132) = 10.20; p < 0.0001	Day 5 p = 0.5729
			Day 6 p = 0.5729
	Week 2		Day 7 p = 0.6598
	interaction	F (6, 108) = 0.9921; p = 0.4344	Day 8 p = 0.9744
	day	F (6, 108) = 1.239; p = 0.3037	Day 9 p = 0.9744
	genotype	F (1, 18) = 0.3597; p = 0.5562	Day 10 p = 0.9744
	subject	F (18, 108) = 30.77; p < 0.0001	Day 11 p = 0.9744
			Day 12 p = 0.9744
			Day 13 p = 0.9744
			Day 14 p = 0.9744
**Number of Runs**	Week 1		Day 1 p = 0.0934
Two-way ANOVA,	interaction	F (6, 132) = 1.664; p = 0.1349	Day 2 p = 0.6731
Holm-Šídák's multiple	day	F (3.049, 67.07) = 1.484; p = 0.2264	Day 3 p = 0.6443
comparisons test	genotype	F (1, 22) = 4.661; ***p = 0.0420***	Day 4 p = 0.5766
	subject	F (22, 132) = 6.068; p < 0.0001	Day 5 p = 0.6731
			Day 6 p = 0.6731
	Week 2		Day 7 p = 0.6443
	interaction	F (6, 108) = 1.215; p = 0.3043	Day 8 p = 0.8495
	day	F (3.403, 61.67) = 4.31; p = 0.0057	Day 9 p = 0.8495
	genotype	F (1, 18) = 0.9097; p = 0.3528	Day 10 p = 0.8495
	subject	F (18, 108) = 25.67; p < 0.0001	Day 11 p = 0.8495
			Day 12 p = 0.8495
			Day 13 p = 0.8495
			Day 14 p = 0.8495
**Total Distance** Simple linear regression	Week 1	F = 4.781, DFn = 1, DFd = 164; ***p = 0.0302***	
	Week 2	F = 1.002, DFn = 1, DFd = 136; p= 0.3185	

Table S5 ***Statistics for Sleep Studies at 8-10 weeks post-injury or sham procedure.*** *Statistical test noted in table.*

**Sleep Studies**	**Main; F, p-value**		**Adjusted p-value**
Sleep assay **Hourly % Sleep over 48 hours** Mixed effects model (REML)	**Sarm1 WT**	**Sham - TBI**	
	time	F (47, 752) = 8.226; p < 0.0001	
	injury	F (1, 16) = 6.675; p = 0.0200	
	interaction	F (47, 752) = 0.9788; p = 0.5151	
	**Sarm1 KO**	**Sham - TBI**	
	time	F (47, 799) = 11.72; p < 0.0001	
	injury	F (1, 17) = 0.02167; p = 0.8847	
	interaction	F (47, 799) = 1.232; p = 0.1408	
	**Shams**	**WT - KO**	
	time	F (47, 705) = 10.50; p < 0.0001	
	genotype	F (1, 15) = 0.9119; p = 0.3548	
	interaction	F (47, 705) = 0.6246; p = 0.9772	
	**TBI**	**WT - KO**	
	time	F (47, 846) = 9.527; p < 0.0001	
	genotype	F (1,18) = 1.029; p = 0.3238	
	interaction	F (47, 846) – 1.319; p = 0.0771	
**Hours Slept: Lights On** Two-way ANOVA, Holm-Šídák's multiple comparisons test	Interaction	F (1, 33) = 1.664; p = 0.2061	WT sham-TBI ***p = 0.0113***
	Injury	F (1, 33) = 12.67; p = 0.0012	KO sham-TBI p = 0.3006
	Genotype	F (1, 33) = 3.188e-006; p = 0.9986	Sham WT-KO p = 0.5739
			TBI WT-KO p = 0.5739
**Hours Slept: Lights Off** Two-way ANOVA, Holm-Šídák's multiple comparisons test	Interaction	F (1, 33) = 0.1857; p = 0.6693	WT sham-TBI p = 0.9605
	Injury	F (1, 33) = 0.4578; p = 0.5034	KO sham-TBI p = 0.8957
	Genotype	F (1, 33) = 1.084; p = 0.3055	Sham WT-KO p = 0.8593
			TBI WT-KO p = 0.9591

REFERENCES

1 Sullivan GM, Knutsen AK, Peruzzotti-Jametti L, Korotcov A, Bosomtwi A, Dardzinski BJ, Bernstock JD, Rizzi S, Edenhofer F, Pluchino Set al (2020) Transplantation of induced neural stem cells (iNSCs) into chronically demyelinated corpus callosum ameliorates motor deficits. Acta Neuropathol Commun 8: 84 Doi 10.1186/s40478-020-00960-3

2 Yu F, Shukla DK, Armstrong RC, Marion CM, Radomski KL, Selwyn RG, Dardzinski BJ (2017) Repetitive Model of Mild Traumatic Brain Injury Produces Cortical Abnormalities Detectable by Magnetic Resonance Diffusion Imaging, Histopathology, and Behavior. J Neurotrauma 34: 1364-1381 Doi 10.1089/neu.2016.4569
